# Non-mass Breast Lesions: Could Multimodal Ultrasound Imaging Be Helpful for Their Diagnosis?

**DOI:** 10.3390/diagnostics12122923

**Published:** 2022-11-23

**Authors:** Wenjuan Guo, Tong Wang, Fan Li, Chao Jia, Siqi Zheng, Xuemei Zhang, Min Bai

**Affiliations:** 1 Department of Ultrasound, Shanghai General Hospital, Shanghai Jiao Tong University School of Medicine, Shanghai 200080, China; 2 Department of Pathology, Shanghai General Hospital, Shanghai Jiao Tong University School of Medicine, Shanghai 200080, China

**Keywords:** non-mass breast lesions, ultrasonography, elasticity, contrast-enhanced ultrasound, multimodal ultrasonic diagnosis

## Abstract

Objective: To develop a prediction model for discriminating malignant from benign breast non-mass-like lesions (NMLs) using conventional ultrasound (US), strain elastography (SE) of US elastography and contrast-enhanced ultrasound (CEUS). Methods: A total of 101 NMLs from 100 patients detected by conventional US were enrolled in this retrospective study. The characteristics of NMLs in conventional US, SE and CEUS were compared between malignant and benign NMLs. Histopathological results were used as the reference standard. Binary logistic regression analysis was performed to identify the independent risk factors. A multimodal method to evaluate NMLs based on logistic regression was developed. The diagnostic performance of conventional US, US + SE, US + CEUS and the combination of these modalities was evaluated and compared. Results: Among the 101 lesions, 50 (49.5%) were benign and 51 (50.5%) were malignant. Age ≥45 y, microcalcifications in the lesion, elasticity score >3, earlier enhancement time and hyper-enhancement were independent diagnostic indicators included to establish the multimodal prediction method. The area under the receiver operating characteristic curve (AUC) of US + SE + CEUS was significantly higher than that of US (*p* < 0.0001) and US + SE (*p* < 0.0001), but there was no significant difference between the AUC of US + SE + CEUS and the AUC of US + CEUS (*p* = 0.216). Conclusion: US + SE + CEUS and US + CEUS could significantly improve the diagnostic efficiency and accuracy of conventional US in the diagnosis of NMLs.

## 1. Introduction

Conventional ultrasound (US), as an invaluable imaging technology without limitation of dense breasts, has been frequently utilized in the clinic to identify or diagnose breast lesions. Breast non-mass-like lesions (NMLs) refer to lesions that lack distinct boundaries on ultrasonography and lack spatial mass effects in two or more scanning directions, accounting for 9.2% of all breast lesions [1]. Several studies have shown that conventional US has a high sensitivity of 95.4% to 100% for detecting breast cancer presenting as NMLs, but specificity is just 6.5% to 42.3% [2,3,4]. The differentiation of NMLs by conventional US remained ambiguous, and there is significant overlap between conventional US characteristics of malignant NMLs and benign NMLs (fibrocystic change, sclerosing adenosis, atypical ductal hyperplasia and intraductal papilloma) [2,5,6,7,8]. These observations underline the importance of correctly identifying and diagnosing breast NMLs detected by conventional US.

More recently, as a supplement to conventional US, US elastography or contrast-enhanced ultrasound (CEUS) has provided extra diagnostic information for breast lesions [9]. Both techniques have unique advantages. Strain elastography (SE) of US elastography can reflect the hardness of the target lesion to enable tissue characterization, and a semi-quantitative method based on a 5-point elasticity scoring system could be used to evaluate the target lesion’s hardness [10]. Contrast-enhanced ultrasound is a non-invasive and effective diagnostic method for the differential diagnosis between benign and malignant breast lesions by identifying dynamic contrast enhancement features that reflect abnormal microvascular perfusion information [11].

Multimodal ultrasound diagnosis is a diagnostic method combining conventional US, US elastography and CEUS. The limited specificity of conventional US might be improved with additional information regarding the elasticity and vascularity of NMLs. To our knowledge, clinical studies of multimodal ultrasound diagnosis for NMLs of the breast are scarce. Hence, the purpose of our study was to explore the imaging characteristics of NMLs in conventional US, SE and CEUS and establish a new prediction model based on multimodal ultrasound imaging to predict the potential malignancy of NMLs.

## 2. Materials and Methods

### 2.1. Patients

From January 2017 to March 2022, 100 patients (the mean age: 51.91 ± 13.68 years; the age range: 26–88 years) with 101 breast lesions (the maximum diameter, 21.05 ± 12.57 mm; range: 4–56 mm) fulfilled the criteria for NMLs detected by conventional US were enrolled in this retrospective study. The exclusion criteria were as follows: (1) age ≤18 years old; (2) pregnancy or breastfeeding; (3) inadequate image data; (4) lack of histopathological confirmation; (5) previous neoadjuvant radiotherapy, chemotherapy, biopsy, or breast surgery. Among these 100 patients, 50 patients presented with a palpable mass, 15 patients presented with nipple discharge and 20 patients complained of pain. Prior to surgical excision, all subjects had received conventional US, SE and CEUS examinations. The pathological results of the specimens obtained by surgery or biopsy were regarded as the reference standard. The interval between the histopathological examination and the ultrasound examination was less than one week.

### 2.2. Ultrasound Examination

Conventional US and SE were performed with the Aplio i900 system (Canon Medical systems Corporation, Otawara, Tochigi, Japan) equipped with a 18LX5 line array probe, and CEUS was performed with the LOGIQ E9 (GE Healthcare, Milwaukee, WI, USA) equipped with a 9L probe. SonoVue (Bracco, Milan, Italy) was used as a contrast agent in the CEUS examinations. Conventional US, SE and CEUS were performed for each lesion. All US scans with the patient in the supine position were performed by either of the two sonographers with 8 and 10 years of experience in breast US, respectively.

Conventional grey-scale and color Doppler US were initially applied to analyze lesion characteristics. The color scale of the Doppler US was preset to a low velocity to capture the intralesional blood-flow signal with minimal background noise. Subsequently, SE images were generated by the same sonographers. Images were displayed in dual mode with the conventional US image on the right and the SE image on the left. To obtain appropriate images, the transducer must be applied with a pressure necessary to maintain contact with the skin, and the square region of interest (ROI) should include the whole lesion and the surrounding tissue. After standard conventional US and SE evaluation, CEUS was performed after a bolus injection of 4.5 mL of contrast medium mixed with SonoVue and saline solution, followed by 5 mL of saline solution. The plane with maximal diameter was chosen as the target plane. A 180-s dynamic image was recorded and saved for further analysis.

### 2.3. Image Analysis

Two radiologists retrospectively reviewed the conventional US, SE and CEUS data, both having more than 10 years of experience in breast US, and they reached a consensus for decisions. Both would have no access to the final histological results and other imaging findings.

The morphological features and blood supply of NMLs were evaluated by conventional US. Their location, maximal diameter, intralesional echo, posterior echo features, orientation, microcalcification inside the lesion, architectural distortion and adjacent ductal changes were recorded. If the calcification diameter was ≤1.0 mm, the calcification was judged to be microcalcification [12]. Because NMLs mainly exhibited ill-defined margins and irregular shapes, the NMLs in our study were categorized as BI-RADS 4a, 4b, 4c and 5 according to the fifth edition of BI-RADS lexicon. The cutoff points of the benign and malignant groups were 4a and 4b. The vascularity of NMLs in color Doppler mode was classified on the basis of Adler’s grade into four categories [13]. In this study, Grade 0 or 1 was considered as scarce vascularity, and Grades 2 or 3 were considered as abundant vascularity.

For SE images, the target lesion was scored as 1 (soft) to 5 (hard) according to the scoring system proposed by Itoh et al. [14]: 1, predominantly green; 2, a mosaic pattern of green and blue; 3, the peripheral part was green and the center was blue; 4, predominantly blue, but its surrounding part was not included and 5, completely blue with its surrounding part. A published study reported that the lesions scored 1–3 were regarded as benign and the lesions scored 4 or 5 were regarded as malignant [15].

The CEUS pattern of each lesion was evaluated. The enhancement indicators of CEUS used for analysis were derived from previous studies and our clinical experience: enhancement time (compared to the surrounding normal breast tissue), enhancement intensity (compared to the surrounding breast tissue at the peak time), enhancement sharpness, enhancement margin, enhancement distribution, enhancement area (compared to that in the grey-scale US) and radial or penetrating vessels.

### 2.4. Statistical Analysis

SPSS 26.0 software (IBM Corporation, Armonk, NY, USA) was used to perform statistical analysis. Quantitative data were expressed as mean ± SD. An independent t-test was used to compare quantitative variables, while the χ2 or Fisher’s exact test was used to evaluate categorical variables. Univariate analysis was used to identify the independent risk factors for NMLs. Features highly relevant to malignancy were included in the multivariate regression analysis. The prediction model was built using the logistic equation. A Hosmer–Lemeshow (H-L) goodness-of-fit test was used to assess the calibration, and *p* ≥ 0.05 was considered well calibrated. The receiver operating characteristic (ROC) curve was constructed to assess the diagnostic performance of the prediction model, the area under the ROC curve (AUC), sensitivity, specificity, accuracy, positive predictive value (PPV) and negative predictive value (NPV) were calculated, with pathological results being used as a reference standard. The ROC curve analysis was conducted to reveal the diagnostic performances of US, US + SE, US + CEUS and US + SE + CEUS, while the Z-test was conducted to compare the AUC values. A *p*-value < 0.05 was considered statistically significant.

## 3. Results

### 3.1. Histopathologic Diagnosis

Among the 101 lesions, 49.5% (50/101) were found to be benign and 50.5% (51/101) malignant. The detailed histopathologic results were presented in Table 1. The mean age of patients with benign and malignant NMLs was 46.40 ± 12.03 years and 57.31 ± 13.13 years, respectively (*p* < 0.001). The mean maximum diameter of benign and malignant NMLs was 18.04 ± 12.89 mm and 24.00 ± 11.63 mm, respectively (*p* = 0.016).

### 3.2. Single Factor Analysis of the Indicators for Malignant NMLs

**Figure 1:**
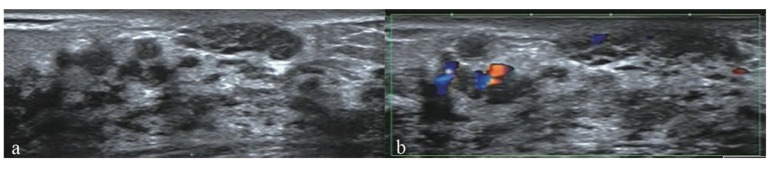
Conventional US images of a 45-year-old female who was diagnosed with DCIS by surgical excision. (**a**) The B-mode US image shows a 37.0 mm non-mass breast lesion with microcalcifications in the outer upper quadrant area next to the nipple of the right breast (arrows); (**b**) The color Doppler US image shows abundant blood supply.

**Figure 2:**
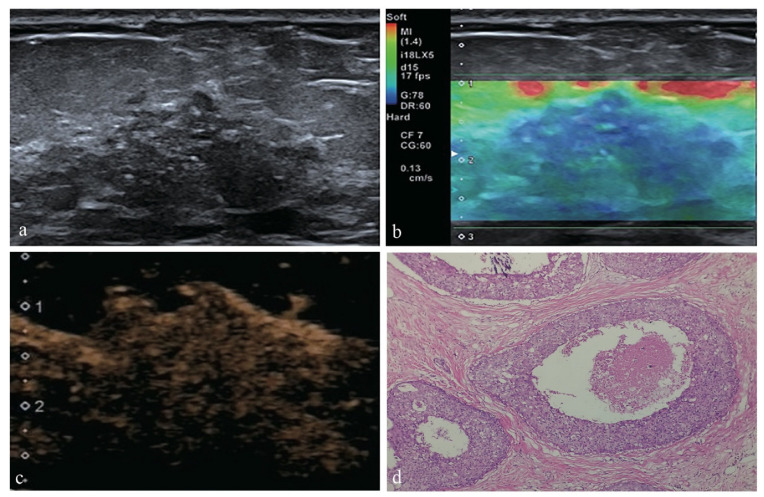
A 58-year-old female was diagnosed with DCIS with histopathology. (**a**) The conventional US image indicated a hypoechoic area at 12 o’clock direction of the left breast with ill-defined margins, irregular shape and microcalcifications; (**b**) The SE image showed more than half of the lesion area was blue with little green spots, scored as 4; (**c**) Under the CEUS pattern, the lesion showed early hyper-enhancement with unclear margin, irregular sharpness, enlarged enhancement area and radial or penetrating vessels; (**d**) Histopathological analysis revealed DCIS (hematoxylin-eosin stain; original magnification, ×100).

The clinical and ultrasound characteristics of NMLs in our study and their correlations with the histopathologic results were outlined in Table 2. The characteristics analysis showed that age ≥45 y, lesion size ≥20, microcalcifications in the lesion, architectural distortion and abundant internal vascularity on conventional US (Figure 1), elasticity score >3 on SE, earlier enhancement time, hyper-enhancement, irregular enhancement sharpness, unclear enhancement margin, enlarged enhancement area and radial or penetrating vessels on CEUS were significantly associated with malignancy (all *p* < 0.05) (Figure 2). None of the factors, such as menstrual history, intralesional echo, posterior echo, orientation, ductal changes, or enhancement distribution, were statistically significant in differentiating benign and malignant breast lesions (all *p* > 0.05).

### 3.3. Developing the Prediction Model

All the features acquired from single factor analysis were included in the regression analysis as independent variables. Then in the multivariate analysis with the stepwise forward variable selection method, all independent risk factors for malignant NMLs were determined in the final step as follows: age ≥45 y (OR: 11.70, *p* = 0.028), microcalcifications in the lesion on conventional US (OR: 12.03, *p* = 0.018), elasticity score >3 on SE (OR: 27.88, *p* = 0.008), earlier enhancement time (OR: 39.95, *p* = 0.006) and hyper-enhancement on CEUS (OR: 13.37, *p* = 0.014).

A logistic regression equation was finally established with the significant predictors as follows: *p* = 1/1 + Exp∑[−7.869 + 2.459 × (if age ≥ 45 y) + 2.487 × (if microcalcifications in the lesion) + 3.328 × (if elasticity score >3) + 3.688 × (if earlier enhancement time) + 2.593 × (if hyper-enhancement)]. The area under the ROC curve (AUC) using this formula was 0.960. With a cutoff value of 0.509, its sensitivity, specificity, PPV, NPV and accuracy were calculated to be 98.0%, 94.0%, 94.3%, 97.9% and 96.0%, respectively.

### 3.4. Comparison of Diagnostic Performance of Different Methods

**Figure 3:**
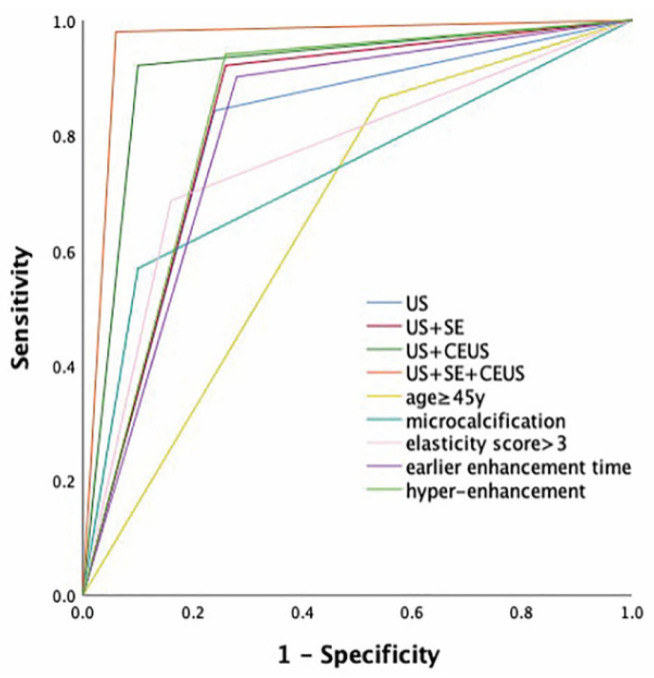
Receiver operating characteristic curves for US, US + SE, US + CEUS, US + SE + CEUS and each of the independent risk factors. The AUC was 0.802 for US, 0.831 for US + SE, 0.911 for US + CEUS and 0.960 for US + SE + CEUS; The AUC was 0.661 for age ≥45 y, 0.734 for microcalcifications, 0.763 for elasticity score >3, 0.811 for earlier enhancement time and 0.841 for hyper-enhancement.

The sensitivity, specificity, PPV, NPV and accuracy of this multimodal diagnostic method for distinguishing between benign and malignant NMLs were summarized in Table 3. Compared with conventional US, US + SE, US + CEUS and US + SE + CEUS all noticeably improved some relevant parameters for diagnosing benign and malignant NMLs. The AUC of US + SE + CEUS was considerably higher than that of US (0.960 vs. 0.802, *p* = 0.002) and US + SE (0.960 vs. 0.831, *p* = 0.008). Furthermore, the AUC of US + SE + CEUS was also higher than that of US +CEUS (0.960 vs. 0.911), but there was no statistical significance (*p* = 0.216) (Figure 3). Thus, both US + SE + CEUS and US + CEUS had better diagnostic efficiency for NMLs.

In addition, the AUC of the prediction model was greater than that of any independent predictor for diagnosing malignant NMLs. The AUC of the prediction model was obviously higher than that of age ≥45 y [0.661 (95% CI, 0.554–0.769)] (*p* < 0.001), microcalcifications in the lesion [0.734 (95% CI, 0.635–0.834)] (*p* < 0.001), elasticity score > 3 [0.763 (95% CI, 0.667–0.859)] (*p* < 0.001), earlier enhancement time [0.811 (95% CI, 0.722–0.900)] (*p* = 0.003) and hyper-enhancement [0.841 (95% CI, 0.758–0.924)] (*p* = 0.013) (Figure 3).

### 3.5. False-Positive and False-Negative Diagnoses with the Multimodal Method

The false-positive rate for the multimodal method was 6.0% (3/50), while the false-negative rate was 2.0% (1/51). The three false-positive NMLs were intraductal papilloma (*n* = 2) and granulomatous mastitis (*n* = 1), in patients ranging in age from 46 to 75, with diameters ranging from 9.5 to 56.0 mm. The false-negative NML was a 55-year-old case of invasive ductal carcinoma, with a diameter of 15.0 mm.

## 4. Discussion

Breast cancer, especially ductal carcinoma in situ (DCIS), can manifest as an NML on ultrasound [16,17,18]. DCIS diagnoses currently account for about 20% of new breast cancer cases in China [19]. In our study, the lesions of breast cancer accounted for 50.5% (51/101) of total NMLs, with DCIS accounting for 33.3% (17/51) of all breast cancer lesions. It is crucial to identify this lesion on the breast US. The combination of conventional US, SE and CEUS may offer a more intuitive and accurate understanding of NMLs. Thus, we conducted this study to develop a multimodal ultrasound prediction model and assess the effectiveness of multimodal ultrasound diagnosis in NML differentiation.

In this study, 18 conventional US, SE and CEUS features as well as clinical characteristics were included as potential predictors for malignancy. The results suggested that age ≥45 y, lesion size ≥20, microcalcifications in the lesion, architectural distortion and abundant internal vascularity on conventional US were detected more frequently in malignant NMLs. The peak age of breast cancer diagnosis in Chinese women is between 45 and 55 [20]. Several studies have reported that patients with breast cancer tend to be older than those with benign lesions [21,22]. In the previous studies [23,24], microcalcifications were identified as an independent risk factor for malignant NMLs, indicating that microcalcifications were related to malignancy. Architectural distortion and ductal changes were also common features of NMLs, but in our study, ductal changes had no meaningful impact on the distinction between malignant and benign NMLs. Besides, despite reports that more than 50% of malignant breast masses exhibit a tendency for longitudinal growth (aspect ratio >1) [25], we discovered that the transverse diameter of 95.0% (96/101) NMLs and 92.2% (47/51) malignant NMLs was parallel to the mammary gland (aspect ratio <1). It may be linked to the fact that malignant NMLs mostly grow along the mammary gland ducts.

Two other studies have identified that malignant NMLs tend to exhibit earlier enhancement time, hyper-enhancement, enlarged enhancement area and radial or penetrating vessels on CEUS [24,26]. In addition to the above enhancement characteristics, one study suggested that malignant NMLs also tended to present unclear enhancement margins and perfusion defects on the contrast-enhanced pattern [27]. Besides, our research has shown that irregular enhancement sharpness was statistically significant (*p* < 0.05) between benign and malignant NMLs. However, contrary to the homogeneous enhancement often observed in benign breast masses [28,29,30,31], 50% (25/50) of benign NMLs displayed heterogeneous enhancement. Loose cell proliferation in a more sclerotic stroma might correlate with heterogeneous enhancement in benign breast lesions [32]. In the clinical work, adenosis and malignant NMLs were considerably similar in morphology, which was the main cause of misdiagnosis. In our study, 94.1% (48/51) of malignant NMLs manifested hyper-enhancement, while 94.4% (17/18) of adenosis manifested hypo-enhancement. On the other hand, 90.1% (46/51) of malignant NMLs manifested earlier enhancement while 88.8% (16/18) of adenosis manifested synchronous enhancement. As a result, enhancement time and intensity were effective characteristics to discriminate between adenosis and malignant NMLs.

Stiffness was another crucial element in the differential diagnosis of benign and malignant lesions. Previous research has shown a strong correlation between the elasticity and the tissue stiffness of benign and malignant breast lesions [33]. In our study, like the diagnosis of mass-like breast lesions by SE, malignant NMLs were stiffer than benign ones. Although DCIS was mostly found to be soft on SE [34], the DCIS of NMLs in our study tended to be harder (11/17). This might be that the semi-quantitative method based on a 5-point elasticity scoring system used to evaluate the target lesion’s hardness was partly subjective. Hence, SWE, a quantitative method, can be added to the multimodal method to increase diagnostic accuracy and sensitivity for DCIS in the future. Besides, granulomatous mastitis often showed heterogeneous hyper-enhancement, enlarged enhancement area, and an unclear enhancement margin on CEUS, which made it difficult to distinguish from malignant NMLs. Some findings have shown that granulomatous mastitis lesions were soft and had low elasticity scores [35,36,37]. Eight cases of granulomatous mastitis were included in this study, and all had an elasticity score ≤3. Therefore, the stiffness detected by SE may play a key role.

The multimodal ultrasound method developed on the logistic regression formula was a more simplified and objective approach with excellent diagnostic efficiency for the differentiation of NMLs. Conventional US provided information about the fundamental characteristics of the lesions, SE revealed details regarding elasticity, and CEUS offered information about microvascular perfusion. In the final, age ≥45 y, microcalcifications in the lesion, elasticity score >3, earlier enhancement time and hyper-enhancement were taken into the formula. In the present study, these parameters were positively correlated with malignancy. We discovered that the combination of conventional US, SE and CEUS improved the performance of conventional US in diagnosing benign and malignant NMLs, with a significant increase in AUC from 0.802 to 0.960 (*p* = 0.002) and also in sensitivity, specificity and accuracy. Additionally, we found that, when compared to US and US + SE, US + SE + CEUS showed the highest diagnostic efficiency, but without statistical difference from US + CEUS. This was different from the results of Zhang et al.’s study which demonstrated that the diagnostic efficiency of US + SE + CEUS was better than US + CEUS [24]. The reason might be that the composition of pathological types and statistical methods in our study were different from those of Zhang et al.’s study.

Using this multimodal method, 97 of 101 NMLs were correctly diagnosed, and 94% (47/50) of benign lesions effectively avoided unnecessary core biopsies, which could relieve patient distress and save medical resources. In addition, the AUC of the multimodal prediction model was higher than that of any independent predictor for diagnosing malignant NMLs. Although the single independent predictor to identify benign and malignant NMLs was more convenient to be used in the clinic than the multimodal prediction model, the latter had greater diagnostic efficacy. In the future, the multimodal method might be employed to discriminate adenosis or granulomatous mastitis from malignant NMLs for clinical utility and reduce biopsy rates after multi-center relevant studies with larger sample sizes.

This study had several limitations. First, it was a retrospective single-center study, and only lesions with complete data were included. Thus, selection and recall biases might exist. Second, there was unavoidable subjectivity in the interpretation of morphological features, blood supply, and enhancement characteristics of NMLs, and only qualitative data of SE and CEUS were included in the study, which might lack quantitative accuracy. Third, the multimodal method was based on the materials we gathered, so its diagnostic performance should be assessed further in prospective studies and in multiple centers.

## 5. Conclusions

Our study indicated that age ≥45 y, microcalcifications in the lesion, elasticity score > 3, earlier enhancement time and hyper-enhancement were independent risk factors for malignant NMLs. The multimodal ultrasound method on the basis of the logistic regression formula and US + CEUS could improve the diagnostic efficiency and accuracy dramatically for the differential diagnosis of NMLs. Although the diagnostic efficacy of US + CEUS + SE was not statistically different from that of US + CEUS, SE examination might be able to give a deeper understanding of the characteristics of NMLs by evaluating the stiffness of the lesion and assist with the subsequent core-needle biopsy of the potential malignant NMLs.

## Figures and Tables

**Table 1:** Histopathological results.

Histopathological Diagnosis	No. of Lesions (*n* = 104)
**Benign lesions**	50
Adenosis	18
Intraductal papilloma	14
Granulomatous mastitis	8
Mammary duct ectasia	5
Fibroadenoma	3
Sclerosing adenosis	2
**Malignant lesions**	51
Ductal carcinoma in situ	17
Invasive ductal carcinoma	16
Invasive ductal carcinoma + ductal carcinoma in situ	9
intraductal papillary carcinoma	4
Solid papillary carcinoma	2
Mucinous breast carcinoma	1
Lobular carcinoma in situ	1
Paget’s disease+ ductal carcinoma in situ	1

**Table 2:** Comparison of clinical information and imaging features between benign and malignant NMLs.

Characteristics	Benign *n* (%)	Malignant *n* (%)	*p*
**Patients**			
Age (years)			<0.001
<45	23(46.0)	7(13.7)	
≥45	27(54.0)	44(86.3)	
Menstrual history			0.051
Menstrual	35(70.0)	26(51.0)	
Menopause	15(30.0)	25(49.0)	
**Conventional US**			
Lesion size (mm)			0.037
<20	30(60.0)	20(39.2)	
≥20	20(40.0)	31(60.8)	
Intralesional echo			0.060
Other echoes	6(12.0)	1(2.0)	
Hypo-echo	44(88.0)	50(98.0)	
Posterior echo			0.092
Other echoes	48(96.0)	43(90.1)	
Attenuation	2(4.0)	8(9.9)	
Orientation			0.362
Parallel	49(98.0)	47(92.2)	
Non-parallel	1(2.0)	4(7.8)	
Microcalcification			<0.001
Absent	45(90.0)	22(43.1)	
Present	5(10.0)	29(56.9)	
Architectural distortion			0.001
Absent	50(100)	40(78.4)	
Present	0(0)	11(21.6)	
Ductal changes			0.145
Absent	35(70.0)	42(82.4)	
Present	15(30.0)	9(17.6)	
Vascularity			0.002
Scarce	44(88.0)	31(60.8)	
Abundant	6(12.0)	20(39.2)	
**SE**			
Elasticity score			<0.001
≤3	42(84.0)	16(31.4)	
>3	8(16.0)	35(68.6)	
**CEUS**			
Enhancement time			<0.001
Synchronous or later	36(72.0)	5(9.8)	
Earlier	14(28.0)	46(90.2)	
Enhancement intensity			<0.001
Iso-/hypo-enhancement	37(74.0)	3(5.9)	
Hyper-enhancement	13(26.0)	48(94.1)	
Enhancement sharpness			<0.001
Regular	23(46.0)	0(0)	
Irregular	27(54.0)	51(100)	
Enhancement margin			0.034
Clear	10(20.0)	3(5.9)	
Unclear	40(80.0)	48(94.1)	
Enhancement distribution			0.373
Homogenous	25(50.0)	21(41.2)	
Heterogeneous	25(50.0)	30(58.8)	
Enhancement area			<0.001
Non-enlarged	40(80.0)	12(23.5)	
Enlarged	10(20.0)	39(76.5)	
Radial or penetrating vessels			0.013
Absent	47(94.0)	39(76.5)	
Present	3(6.0)	12(23.5)	

**Table 3:** Diagnostic performance of US, US + SE, US + CEUS and US + SE + CEUS.

	Sensitivity (%)	Specificity (%)	PPV (%)	NPV (%)	Accuracy (%)	AUC (95% Confidence Interval)
US	84.3	76	78.2	82.6	80.2	0.802
	(43/51)	(38/50)	(43/55)	(38/46)	(81/101)	(0.711–0.892)
US + SE	92.2	74	78.3	90.2	83.2	0.831
	(47/51)	(37/50)	(47/60)	(37/41)	(84/101)	(0.746–0.916)
US + CEUS	92.2	90	90.4	91.8	91.1	0.911
	(47/51)	(75/50)	(47/52)	(45/49)	(92/101)	(0.846–0.975)
US + SE + CEUS	98	94	94.3	97.9	96	0.96
	(50/51)	(47/50)	(50/53)	(47/48)	(97/101)	(0.916–1.000)

PPV, positive predictive value; NPV, negative predictive value.

## Data Availability

Research data are available on request from the corresponding author.
